# Non-Restoring Array Divider Using Optimized CAS Cells Based on Quantum-Dot Cellular Automata with Minimized Latency and Power Dissipation for Quantum Computing

**DOI:** 10.3390/nano12030540

**Published:** 2022-02-04

**Authors:** Hyun-Il Kim, Jun-Cheol Jeon

**Affiliations:** 1 Department of Robotics Engineering, Daegu Gyeongbuk Institute of Science & Technology, Dalseong-gun, Daegu 42988, Korea; hyunil89@dgist.ac.kr; 2 Department of Convergence Science, Kongju National University, Gongju 32588, Korea

**Keywords:** nanotechnology, quantum simulation, quantum-dot cellular automata, non-restoring array divider, public-key cryptography

## Abstract

Many studies have addressed the physical limitations of complementary metal-oxide semi-conductor (CMOS) technology and the need for next-generation technologies, and quantum-dot cellular automata (QCA) are emerging as a replacement for nanotechnology. Meanwhile, the divider is the most-used circuit in arithmetic operations with squares and multipliers, and the development of effective dividers is crucial for improving the efficiency of inversion and exponentiation, which is known as the most complex operation. In most public-key cryptography systems, the corresponding operations are used by applying algebraic structures such as fields or groups. In this paper, an improved design of a non-restoring array divider (N-RAD) is proposed based on the promising technology of QCA. Our QCA design is focused on the optimization of dividers using controlled add/subtract (CAS) cells composed of an XOR and full adder. We propose a new CAS cell using a full adder that is designed to be very stable and compact so that power dissipation is minimized. The proposed design is considerably improved in many ways compared with the best existing N-RADs and is verified through simulations using QCADesigner and QCAPro. The proposed full adder reduces the energy loss rate by at least 25% compared to the existing structures, and the divider has about 23%~4.5% lower latency compared to the latest coplanar and multilayer structures.

## 1. Introduction

The progress of miniaturization in complementary metal-oxide semiconductor (CMOS) technology faces physical limitations such as short channel effects and high-power dissipation [1]. One of the most promising nanotechnologies is quantum-dot cellular automata (QCA), which was initially proposed by C.S. Lent et al. in the early 1990s [2]. Since then, a significant amount of research has focused on QCA, both theoretically and experimentally. They have become a promising candidate for use in nano-computing.

The fundamental component of circuit execution is a QCA cell that is extremely compact, and therefore facilitates extreme densities. Each technical and exploratory investigation on QCA determines which QCA circuits can perform at high operating frequency wavelengths using minimal energy expenditure [3,4]. QCA technology is the most favorable among evolving nanotechnologies. Unlike current switching semiconductor technology, QCA encode binary information using electron positions in square cells. The cells each comprise four quantum dots and two mobile electrons; the electrons are always arranged diagonally in the cells owing to Coulombic repulsion. Thus, two possible configurations, polarization (−1) and (+1), can be created. The function of switching and power gain to the circuits is provided by a QCA clock [2,3,4,5].

QCA can be used to implement various existing digital logic circuits. Combination circuits such as multiplexers and full adders and sequential circuits such as flip-flops, counters, and memories can all be easily implemented visually in accordance with quantum logic [6,7,8,9,10]. Above all, if the structure of an operator such as adder, subtractor, multiplier, divider, and inverter increases, delay time, space complexity, and energy dissipation must be seriously considered in circuit configuration [11,12,13,14,15,16,17,18,19,20,21].

Divider operations, which are the most complex of the basic arithmetic operations, play an important role in the design of digital processors. Many algorithms are used for implementing divider operations, such as the restoring, non-restoring, Goldschmidt, Sweeney–Robertson–Tocher (SRT) and Newton–Raphson division algorithms [11]. Among them, the restoring and non-restoring algorithms are simple in implementation and based on addition and subtraction, which are more suitable for integrated circuit design. This paper deals with non-restoring array dividers (N-RAD) because they are considerably more efficient and faster than regular RADs. More importantly, N-RADs have a highly regular structure, and each cell only needs to connect to the nearest neighboring cells, which makes them highly efficient for hardware design. The contribution of this work can be itemized as follows.

- We propose a full adder with minimized area and latency. Using this, a controlled add/subtract (CAS) cell and divider are proposed.
- Through operation analysis and comparison through circuit simulation, the delay time, which is the most important consideration in mid-to-large circuit designs such as dividers, is optimized.
- The energy loss of the entire structure is minimized by minimizing the energy dissipation of the full adder, which is the main operation structure of the divider.

In this paper, we propose a 3 × 3 N-RAD composed of cells using full adders and XOR gates. This paper is organized as follows. Section 2 introduces the basic concepts related to QCA and N-RADs. The basic unit of the proposed design is a specific cell interaction principle-based structure that is used as a three-input XOR gate [3]. A one-bit full adder is designed using the XOR gate, and a basic unit of N-RAD architecture is designed in a single layer with a robustness feature called a controlled add/subtract cell. Finally, a QCA design for N-RADs is developed with a 3 × 3-bit example considering scalability features. It is easy to expand this design to *n × n*-bit. We discuss the proposed QCA designs in detail in Section 4 with structural and power dissipation analysis. In particular, the simulation results and comparisons between the proposed designs and the existing ones are presented. Finally, we provide conclusions in Section 5.

## 2. Related Works

This section gives a basic explanation of QCA, the operation principles of the XOR gates using cell interaction, and binary division using N-RADs.

### 2.1. Basic QCA

A basic QCA cell is implemented through a quadratic-shaped cell that includes four quantum dots at the corners of the cell. These quantum dots are also known as potential wells. Each quantum dot has several nanometers, which are capable of deceiving the electrons inside these wells. Special means-tunneling junctions connect these four quantum dots with each other, and the electrons can tunnel between quantum dots by breaking the barrier. The tunneling period is controlled by a clock signal. Each electron can move freely within the cell boundaries, but it cannot leave the cell [12,13,14,15,16].

**Figure 1:**
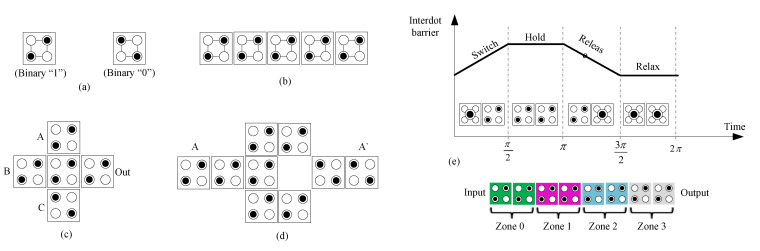
QCA basics: (**a**) regular cells, (**b**) rotated cells, (**c**) majority gate, (**d**) robust inverter, and (**e**) QCA clocking.

The electrons occupy diagonal positions in the cell because of the mutual repulsive force between the two electrons. Therefore, in this case, only two electron adjustments are possible, and these two adjustments are denoted by binary 0 and binary 1. Binary information is represented by the cell polarization (P), i.e., “P = +1.0” means “binary 1”, and “P = −1.0” means “binary 0”, as shown in Figure 1a. A wire is implemented in QCA by allocating cells side by side in a series, as shown in Figure 1b.

The basic elements of QCA are the inverter and three-input majority gate. The behavior of all logic gates in the QCA circuit is based on the majority gate. It is built using five standard cells, as shown in Figure 1c. A device cell placed at the center plays the main role in determining the results of the gate, and the other four cells that cover the four sides of the device cell are input and output cells. A two-input AND and OR gate is implemented by fixing logic “0” and “1”, respectively, at one of the majority gate’s inputs. The majority gate follows the equation *M* (*A*, *B*, *C*) = *AB* + *BC* + *AC*. The inverter that makes the input value the opposite value can be configured in various forms, and Figure 1d shows the inverter with the strongest signal strength.

The QCA clock mechanism plays a very important role in the QCA circuit’s design [22]. Firstly, it provides QCA circuits with necessary power, and it is also used in pipelining data propagation. The circuit is divided into four clock zones (Zones 0–3) and these zones are driven by four-phase clock signals, as shown in Figure 1e. Using (π/2) phase-shifted signals, each clock zone has one of four phase states among Switch, Hold, Release, and Relax. Computation begins during the Switch state and holds the polarization during the Hold state. During the Release and Relax states, the QCA cell is prepared for the next computation.

### 2.2. Cell Interaction XOR Gate

The gate level of the cell interaction exclusive-OR (CIXOR) gate is used to obtain the full adder for implementing CAS cells [3]. After designing the CAS cell, we implement the N-RAD architecture to form a QCA array divider. Common QCA XOR gates are designed by using the AND-OR-INVERTER method defined as follows:

(1) $$A⨁B=\bar{A}B+A\bar{B}=M\left(M\left(\bar{A},\;B,\;0\right)+M\left(A,\bar{B},0\right),1\right)\;A⨁B⨁C=ABC+\bar{AB}C+A\bar{BC}+\bar{A}B\bar{C}=M\left(M\left(\bar{A},\;B,\;C\right)+M\left(A,\bar{B},C\right),\bar{C}\right)$$

**Figure 2:**
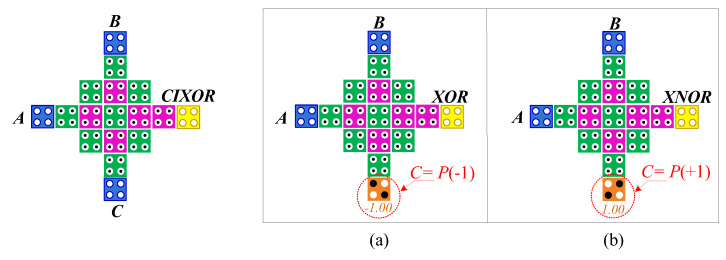
CIXOR gate design: (**a**) XOR gate and (**b**) XNOR gate.

As shown in Equation (1), two-input and three-input XOR gates require five (three majority gates and two inverters) gates and six (three majority gates and three inverters) gates, respectively. However, the CIXOR structure is only a gate that is based on the direct cell interaction principle and the QCA clock mechanism. It has three inputs and one output, and it yields a three-input XOR output as a result. Furthermore, two-input logic XOR or XNOR gates can be easily implemented by setting the third input of the CIXOR gate to “0” or “1”. Figure 2 shows that the CIXOR gate can be used as both types of gates depending on whether the input value, C, is P(−1) or P(+1).

### 2.3. Non-Restoring Array Divider

Binary division is basically a procedure for determining the number of times the divisor *Y* divides the dividend *N*, which results in the quotient “*q*”. At each stage of the process, the divisor *Y* divides *N* into a group of bits. The divisor divides the bit group when the divisor value is less than or equal to the value of these bits. Therefore, the quotient is 1 or 0. Here, the N-RAD process is shown in the following Equations:

(2) $$q_{i+1}=\{\begin{array}{c} 1,\;\;if\;\;\;\;R_{i}>0\\ \;0,\;\;if\;\;\;\;R_{i}<0 \end{array}$$

(3) $$R_{i+1}=\{\begin{array}{c} \;2R_{i}-Y,\;\;if\;\;\;\;R_{i}>0\\ \;\;2R_{i}+Y,\;\;if\;\;\;\;R_{i}<0 \end{array}$$

(4) $$r=\{\begin{array}{c} \;2^{-n}\cdot R_{n},\;\;if\;\;\;\;R_{i}>0\\ \;\;\;2^{-n}\cdot (R_{n}+Y),\;\;if\;\;\;\;R_{i}<0 \end{array}$$

where *i* represents the recursion index, partial remainder *R_i_* is a remainder in the *i^th^* iteration, the quotient is *q*, the divisor is *Y*, and the final remainder is *r*. A basic N-RAD cell, called a controlled add/subtract (CAS) cell, consists of a full adder and an XOR gate, as well as the controlled input *P*. The divisor input is forwarded to the full adder via the XOR gate, and the function of the CAS cell (addition or subtraction) is controlled by the input *P*. The CAS cell reads the practical remainder from the previous stage, and depending on the quotient of the last stage, it adds the divisor to obtain the remainder for the next stage [16,17].

In the literature, there have been several prior works on the implementation of QCA-based N-RADs with different techniques [17,18,19,20,21]. For example, the N-RAD divider in [17] was implemented with a single layer using a clock phase-based wire crossing. However, the propagation delay of the final divider increased due to the QCA crossover technique. The scalability of the design was also not considered well. N-RADs in [18,19] were designed as a multilayer structure based on majority gates and inverters. Recently, a new technical design work has been presented using a cell interaction-based XOR gate in [20,21]. However, these studies also have some limitations in implementation, because propagation delay throughout the wire was not well-considered. Here, we present an implementation of N-RADs using our cell interaction principle-based XOR gate.

## 3. Proposed Structures

In this section, we propose an N-RAD using a proposed CAS cell which is composed of a CIXOR gate and a full adder.

### 3.1. Proposed CAS Cell

**Figure 3:**
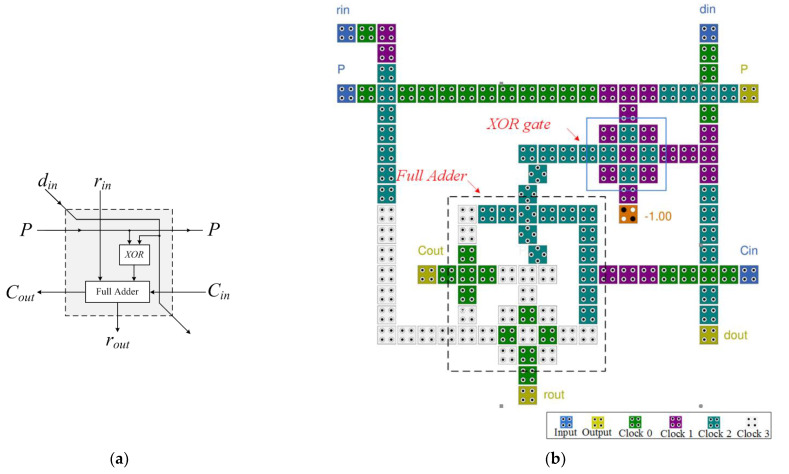
CAS cell: (**a**) block diagram and (**b**) QCA circuit.

The basic unit of the N-RAD is the CAS cell. There are four inputs, *d_in_*, *r_in_*, *C_in_*, and *P*, as well as two outputs, *r_out_* and *C_out_*, as depicted in Figure 3. The function of the CAS cell is defined by the following Equations:

(5) $$r_{out}=r_{in}⨁(d_{in}⨁P)⨁C_{in}\;C_{out}=r_{in}\left(d_{in}⨁P\right)+r_{in}C_{in}+\left(d_{in}⨁P\right)C_{in}$$

Figure 3a shows a block diagram of the CAS cell, which is built using a full adder and an XOR gate. In the structure, *r_in_*, *d_in_*, *C_out_*, and *r_out_* represent the dividend, divisor, quotient, and reminder, respectively. The proposed full adder for the CAS cell is also implemented using the CIXOR gate, as indicated by the dashed square in Figure 3b. The output of the proposed CAS cell design is generated after 1.25 clock cycles. The QCA layout of the CAS cell consumes less latency as it uses a CIXOR gate and clock phase-based crossover.

### 3.2. Design and Implementation of N-RAD

**Figure 4:**
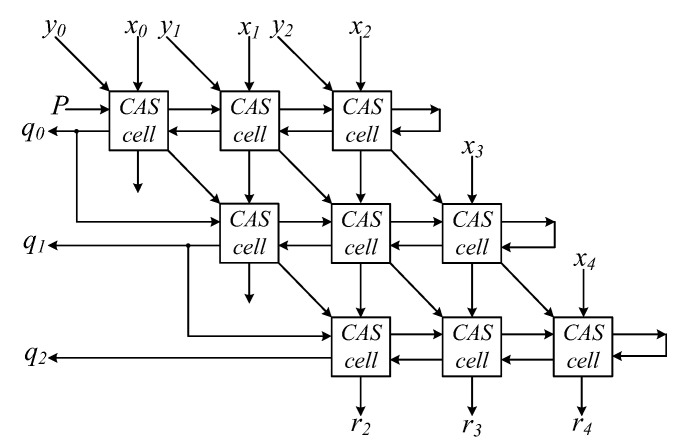
Block diagram of 3 × 3 N-RAD.

Figure 4 shows the block diagram of the 3 × 3 N-RAD architecture with a 4-bit dividend (*x*_1_, *x*_2_, *x*_3_, *x*_4_) and 2-bit divisor (*y*_1_, *y*_2_). A positive number is represented by assigning ”0“ to the first bit of both the dividend (*x*_0_) and divisor (*y*_0_) as their signs. The inputs at the top and right edge of the array import the 2-bit divisor and 4-bit dividend, respectively. The output on the left side of the array produces a 3-bit quotient (*q*_0_, *q*_1_, *q*_2_), and then each quotient bit is propagated to the next row as the control signal *P*. The outputs at the bottom of the array produce a 3-bit final reminder (*r*_2_, *r*_3_, *r*_4_).

**Figure 5:**
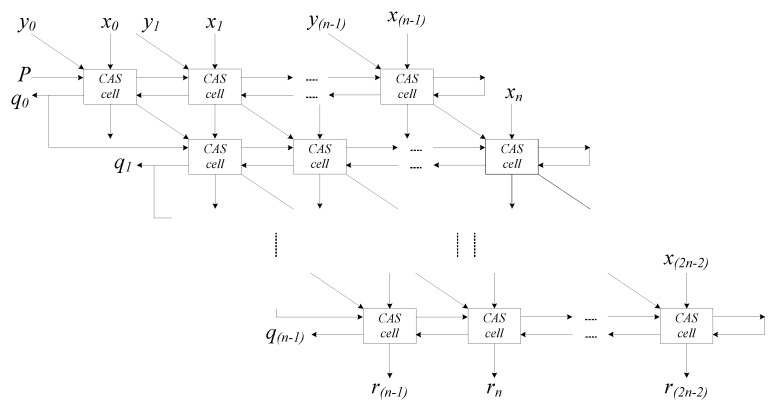
Block diagram of *n × n* N-RAD.

Generally, the logic circuit in QCA is designed using a majority gate and inverter because it is the basic unit of QCA technology. First, the Boolean function of the combinational circuit is converted into its equivalent majority logic expression, and then the architecture is designed according to this logic expression. However, in some cases, the circuit requires more gates compared to its original Boolean function. In fact, the number of gates is the most important factor affecting the performance of the circuit as they determine the complexity and latency of the circuit. Considering these aspects, the QCA design of the N-RAD is implemented using the proposed CIXOR gate. As a result, the numbers of gates used is reduced. The proposed design of the QCA layout is shown in Figure 5.

**Figure 6:**
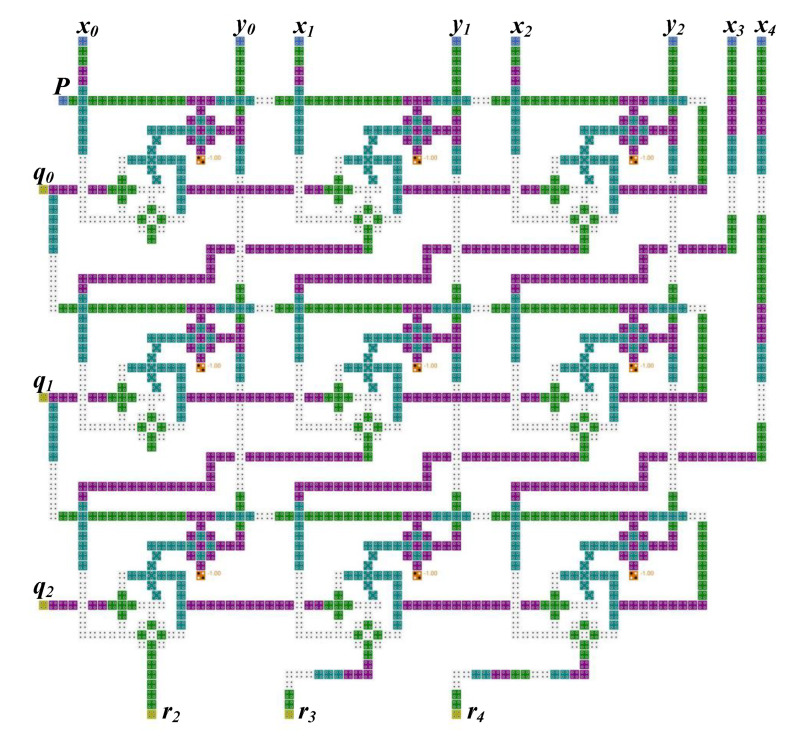
Proposed 3 × 3 N-RAD design using QCA.

Each CAS cell consumes a delay of 1.25 clock cycles to obtain a reliable output. Furthermore, it is important to consider the scalability aspect of the architecture. Therefore, we designed a 3 × 3-bit array divider considering these features. In fact, the *n* × *n*-bit divider is formed by adding *n*^2^ CAS cells to the regular array, as shown in Figure 6. Here, we only present the *n* = 3 form for simplicity; however, it is easy to expand it to *n* bits. A clock phase-based logical crossover technique is used to cross the wires throughout the QCA layout owing to the noise stability problem.

## 4. Discussion

### 4.1. Structural Analysis

We simulated the proposed designs using the QCADesigner (version 2.0.3) tool [22], which has been used for various structures [23,24,25,26,27,28], and obtained stable and reliable simulation results. There are two different simulation (bistable approximation and coherence vector) engines that are used to simulate QCA circuits. In this study, we used both engines, and the parameters are given in Table 1.

**Figure 7:**
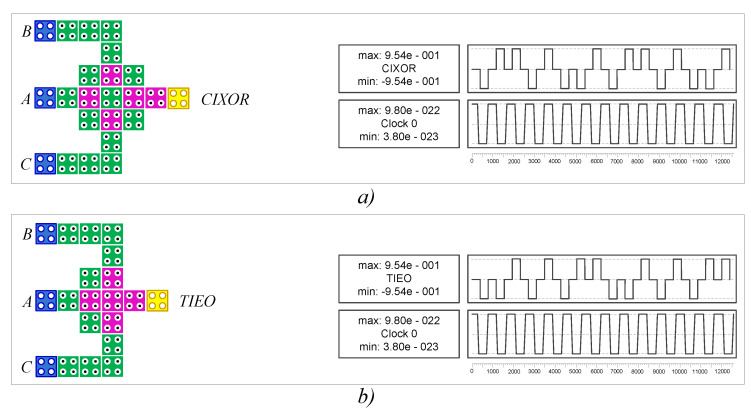
Robustness of simulation results: (**a**) CIXOR gate and (**b**) TIEO gate.

Recently introduced previous works in [20,21] used a three-input Exclusive-OR (TIEO) gate [12] to efficiently implement N-RAD designs. However, the robustness of this TIEO gate is not high enough. For example, if the incoming signals are parallel to the input lines of the TIEO gate, the result may unexpectedly change. The CIXOR gate shows a reliable and stable result, and the verification of both gates is depicted in Figure 7a,b, respectively.

**Figure 8:**
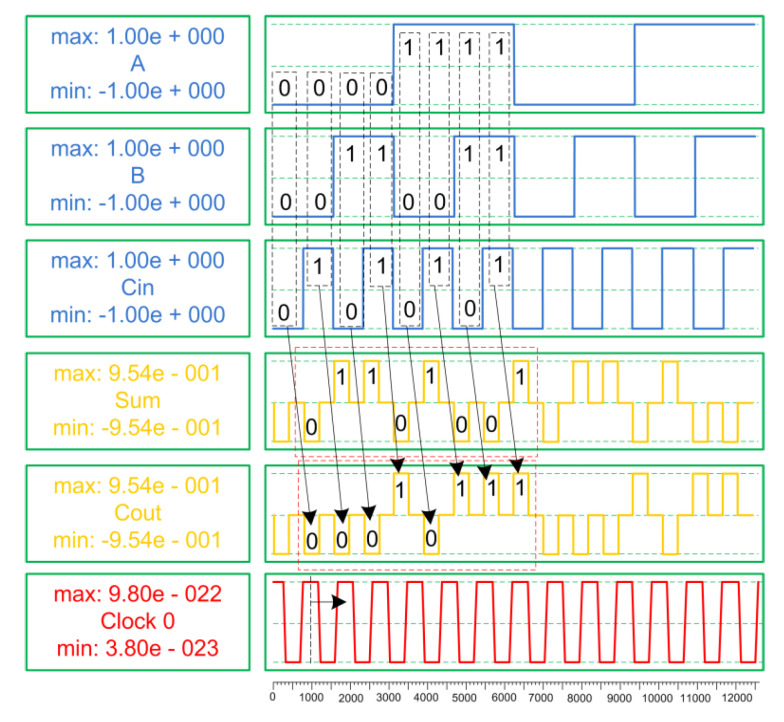
Simulation results of the proposed full adder.

**Figure 9:**
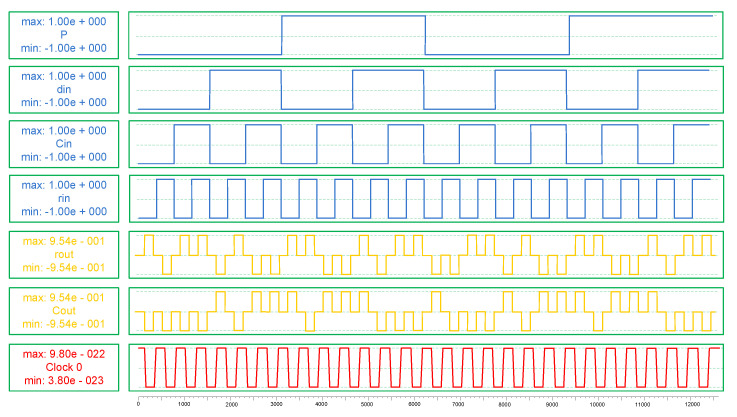
Simulation results of CAS cell.

Comparison tables show the results obtained from the QCA implementations (CIXOR, full adder, and CAS cell) for the N-RAD architecture in terms of complexity, area, and latency factors. Firstly, we conducted structural analysis between the TIEO and CIXOR gates because they are the basic building blocks in efficient N-RAD design. Table 2 shows that these XOR function structures are almost the same in terms of their hardware complexity; however, the CIXOR gate is more robust compared to the TIEO gate. Second, we conducted structural analysis among full adders because they are the main part of the CAS cell. Therefore, the obtained simulation results are reliable and stable, as shown in Figure 8 and Figure 9.

In any case, the *AT*^2^ (area × time^2^) method, which has been used most recently, was adopted for a clearer comparison of the proposed structure with other models [27,28,29,30]. It is gaining much attention as a realistic comparison logic that emphasizes the importance of time rather than area. As a result, as shown in Table 3, it can be confirmed that the proposed FA shows the best performance compared to the previous structures of both coplanar and multilayer full adders. Additionally, in the N-RAD construction period, the pipelining technique in the proposed CAS cell assists it to achieve fewer delays than the best existing ones. A comparison of QCA specifications among designs for the divider is given in Table 4, and the proposed 3 × 3-bit QCA divider performs better than all of the prior best designs [20,21] in terms of latency.

Compared with the best recent structures, our proposed full adder has optimized costs in both area and latency, as shown in Table 3. The single-layer structure in [12] has almost a similar cost to ours, but the structure is only designed to optimize the full adder and not to design the divider. Nevertheless, our circuit shows an improvement of 20% compared to that in *AT*^2^ analysis. As a result, the input and output lines are divided into two, which reduces connectivity and scalability with other circuits. The proposed structure is designed with one input and one output in each direction to make the divider, and the connectivity with other circuits is very easy.

As shown in Table 4, the comparison of the N-RAD structure is based on the total area used to design the corresponding circuit, the latency from the input to making the output, and the circuit design structure. In this test, for a fair comparison, we did not concern ourselves with the input *P* since it can technically be located in several places. In 2018, a type of N-RAD with good complexity was proposed in [30], but it does not work, so we do not mention it here. Meanwhile, the N-RADs in [19] and [21] are designed with a multilayer structure so that they can be easily reduced in area and latency, so they cannot become legitimate opponents of our design; however, it is observable that the proposed design has the best results in terms of latency.

### 4.2. Power Dissipation Analysis

Furthermore, power dissipation was also calculated for the proposed full adder and compared with existing ones [17,31,32,33,34] for three different tunneling energy levels (0.5*E_k_*, 1.0*E_k_*, and 1.5*E_k_*) at temperatures of 2*K*. In order to estimate the energy dissipation of the QCA circuit, the QCAPro [35] tool was used. Energy dissipation is calculated at three different tunneling energy levels (0.5*E_k_*, 1.0*E_k_*, and 1.5*E_k_*) at temperatures of 2K. Specifically, the dissipated energy of the whole circuit for each input combination was evaluated by the tool at various tunneling energy levels based on non-adiabatic switching.

**Figure 10:**
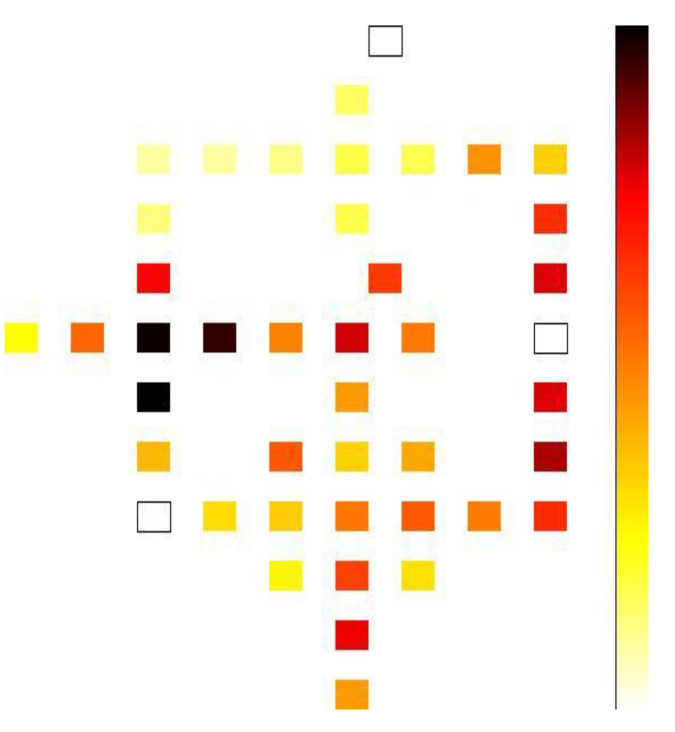
Power dissipation map of the full adder at a temperature of 2*K* and tunneling energy of 0.5*E*_k_.

A power dissipation map of our proposed full adder at a temperature of 2K with 0.5*E_k_* is shown in Figure 10. This indicates that the darker cell in the circuit dissipates more energy than others. Moreover, to obtain a clear picture the performance of power dissipation for the full adders, a graphical comparison was conducted, as shown in Table 5. It can be clearly seen that the proposed full adder made significant achievements. We have reduced more than 25% of the average amounts of energy dissipation presented in Table 5.

In this study, we could not compare energy dissipation for the dividers since it is impossible to measure using existing simulators. However, it is easy to predict that the proposed N-RAD has a lower energy loss rate than a circuit designed with a multilayer structure. Energy loss often occurs at intersections, and the interlayer distance of a multilayer structure is closer than the distance between cells of a coplanar structure, so it is common for them to show a lot of power dissipation.

## 5. Conclusions

In this paper, we propose a 3 × 3-bit N-RAD based on QCA technology. N-RADs are more advantageous than RADs and they are the best option for large data calculations. Our N-RAD was built using CAS blocks in a pipelined style for easy control. Furthermore, compared to RADs, it has low amounts of complexity in most aspects. Thus, N-RADs are more suitable for large-operand-size computation. We proposed the best full adder and divider with a cell interaction XOR gate. The results show that our structures have the best *AT*^2^ complexity or latency with excellent connectivity and scalability in addition to a small amount of power dissipation. The proposed full adder showed a performance of at least 25% improvement compared to the existing structures in all fields of *AT*^2^ complexity and energy loss. The divider also showed the lowest latency, and it is expected that the energy loss was also optimized. In future research, we will design the optimal arithmetic unit including squares, multipliers, and dividers by various designs and experiments on both single layer and multilayer structures, and enhance the QCAPro to produce the result of energy loss. In addition, the proposed operators will be applied to algebraic structures such as fields or groups to be used for cryptographic operator implementation or cryptanalysis.

## Figures and Tables

**Table 1:** Simulation Parameters.

Parameters	Bistable Approximation	Coherence Vector
Cell size (nm)	18	18
Dot diameter (nm)	5	5
Cell separation (nm)	2	2
Layer separation (nm)	11.5	11.5
Clock high (J)	9.8 × 10^−22^	9.8 × 10^−22^
Clock low (J)	3.8 × 10^−23^	3.8 × 10^−23^
Clock shift	0	0
Clock amplitude factor	2.0	2.0
Relative permittivity	12.9	12.9
Radius of effect (nm)	65	80
Number of samples	50,000	-
Convergence tolerance	0.001	-
Max. iterations per sample	100	-
Temperature (K)	-	1
Total simulation time (s)	-	7 × 10^−11^

**Table 2:** Comparison between TIEO and CIXOR gates.

Circuit	Cell Count	Total Area (Μm^2^)	Latency (Clock Cycle)	Condition
TIEO gate [12]	14	0.02	0.50	Normal
CIXOR gate	17	0.02	0.50	Robust

**Table 3:** Comparison of full adders.

Circuit	Cell Count	Total Area (μm^2^)	Latency (Clock Cycle)	Crossover	*AT* ^2^
[17]	111	0.20	1.25	Coplanar	0.312500
[18]	94	0.14	0.75	Multilayer	0.078750
[19]	58	0.03	0.75	Multilayer	0.016875
[20]	43	0.05	0.75	Coplanar	0.028125
[21]	62	0.05	0.70	Multilayer	0.024500
[12]	41	0.06	0.50	Coplanar	0.015000
Proposed	42	0.05	0.50	Coplanar	0.012500

**Table 4:** Comparison of the N-RADs.

Circuit	Cell Count	Total Area (μm^2^)	Latency(Clock Cycle)	Structure
[17]	3742	6.20	26.25	Coplanar
[20]	1686	3.34	6.75	Coplanar
[19]	1852	1.92	7.50	Multilayer
[21]	1436	1.53	5.75	Multilayer
The proposed	1489	2.10	5.50	Coplanar

**Table 5:** Power dissipation analysis of full adders.

Circuit	Avg. Leakage Energy Dissipation (meV)			Avg. Switching Energy Dissipation (meV)			Avg. Energy Dissipation of Circuit (meV)		
	0.5*E_k_*	1.0*E_k_*	1.5*E_k_*	0.5*E_k_*	1.0*E_k_*	1.5*E_k_*	0.5*E_k_*	1.0*E_k_*	1.5*E_k_*
[17]	48.69	150.52	270.62	269.26	233.79	199.82	317.95	384.31	470.44
[31]	27.69	84.08	151.39	159.68	140.88	122.21	187.37	224.96	273.60
[32]	27.91	85.42	153.58	147.67	127.78	108.61	175.58	213.20	262.19
[33]	40.07	118.85	208.7	181.61	153.67	128.82	221.68	272.52	337.53
[34]	37.21	98.5	165.04	61.97	50.90	42.31	99.19	149.40	207.34
Proposed	22.71	62.93	107.72	62.60	52.94	44.42	85.31	115.87	152.13

## Data Availability

The data presented in this study are available on request from the authors.
